# A comprehensive multi-omics approach uncovers adaptations for growth and survival of *Pseudomonas aeruginosa* on *n*-alkanes

**DOI:** 10.1186/s12864-017-3708-4

**Published:** 2017-04-28

**Authors:** Sarah L. Grady, Stephanie A. Malfatti, Thusitha S. Gunasekera, Brian K. Dalley, Matt G. Lyman, Richard C. Striebich, Michael B. Mayhew, Carol L. Zhou, Oscar N. Ruiz, Larry C. Dugan

**Affiliations:** 1 Biosciences and Biotechnology Division, Physical and Life Sciences Directorate, Lawrence Livermore National Laboratory, Livermore, CA 94550 USA; 2 Environmental Microbiology Group, University of Dayton Research Institute, University of Dayton, Dayton, OH 45469 USA; 3 Huntsman Cancer Institute, University of Utah School of Medicine, Salt Lake City, UT 84112 USA; 4 Computational Engineering Division, Lawrence Livermore National Laboratory, Livermore, CA 94550 USA; 5 Computing Applications and Research Department, Global Security Computing and Applications Division, Lawrence Livermore National Laboratory, Livermore, CA 94550 USA; 6 Fuels and Energy Branch, Aerospace Systems Directorate, Air Force Research Laboratory, Wright-Patterson AFB, OH 45433 USA

**Keywords:** *Pseudomonas aeruginosa*, Multi-omics, Ribosome footprinting, Quorum sensing, Alkane degradation

## Abstract

**Background:**

Examination of complex biological systems has long been achieved through methodical investigation of the system’s individual components. While informative, this strategy often leads to inappropriate conclusions about the system as a whole. With the advent of high-throughput “omic” technologies, however, researchers can now simultaneously analyze an entire system at the level of molecule (DNA, RNA, protein, metabolite) and process (transcription, translation, enzyme catalysis). This strategy reduces the likelihood of improper conclusions, provides a framework for elucidation of genotype-phenotype relationships, and brings finer resolution to comparative genomic experiments. Here, we apply a multi-omic approach to analyze the gene expression profiles of two closely related *Pseudomonas aeruginosa* strains grown in *n*-alkanes or glycerol.

**Results:**

The environmental *P. aeruginosa* isolate ATCC 33988 consumed medium-length (C_10_–C_16_) *n*-alkanes more rapidly than the laboratory strain PAO1, despite high genome sequence identity (average nucleotide identity >99%). Our data shows that ATCC 33988 induces a characteristic set of genes at the transcriptional, translational and post-translational levels during growth on alkanes, many of which differ from those expressed by PAO1. Of particular interest was the lack of expression from the *rhl* operon of the quorum sensing (QS) system, resulting in no measurable rhamnolipid production by ATCC 33988. Further examination showed that ATCC 33988 lacked the entire *lasI*/*lasR* arm of the QS response. Instead of promoting expression of QS genes, ATCC 33988 up-regulates a small subset of its genome, including operons responsible for specific alkaline proteases and sphingosine metabolism.

**Conclusion:**

This work represents the first time results from RNA-seq, microarray, ribosome footprinting, proteomics, and small molecule LC-MS experiments have been integrated to compare gene expression in bacteria. Together, these data provide insights as to why strain ATCC 33988 is better adapted for growth and survival on *n*-alkanes.

**Electronic supplementary material:**

The online version of this article (doi:10.1186/s12864-017-3708-4) contains supplementary material, which is available to authorized users.

## Background

“Omic” technologies have advanced significantly in the years since the introduction of microarrays, and continuous improvements to sample processing speed, coupled with the decreasing cost of deep sequencing, have made these techniques accessible to a wider audience. Microarrays are now often supplemented or replaced by RNA-seq experiments, and tools like proteomics, ribosome profiling/Ribo-seq and metabolomics are becoming commonplace. These individual techniques are data-intensive and holistic, which make them more useful than traditional molecular biology techniques when it comes to probing a complex system [1]. However, even with these advantages, single datasets still offer only one dimension of an organism’s activities. It is a well-studied phenomenon, for example, that mRNA levels often correlate poorly with protein levels, especially in eukaryotes [2–4]. Even in bacteria, where many genes and operons are primarily regulated at the transcriptional level, newer research shows that co- and post-transcriptional regulatory networks have an important role in the expression of metabolic genes and protein abundance [5, 6]. Additionally, no matter the model system, transcription measurements alone provide little to no information about the catalytic activity of enzymes. For these reasons, researchers must collect and analyze multiple layers of -omics data simultaneously to truly understand a complex biological system. This data integration process, however, represents a major challenge for the systems biology field [7].

To test the hypothesis that a multi-omics approach would provide a more fully realized model of a biological system, we collected and integrated RNA-seq, microarray, Ribo-seq, proteomic and small molecule data over the course of a single experiment. As our test system, we examined degradation of normal alkanes by two related strains of the bacterium *Pseudomonas aeruginosa*, as the basic steps in this process are understood, but a system-wide analysis had not yet been performed. Here, we show for the first time, that integration of a unique combination of -omic data sets uncovers novel insights into a complex biological system.

## Results

### Two strains of *Pseudomonas aeruginosa* show divergent gene expression profiles during growth on *n*-alkanes

Multiple *Pseudomonas aeruginosa* strains, including the clinical isolate PAO1, have been shown to grow on hydrocarbons as a sole carbon source [8–11]. Normal alkanes (*n*-alkanes) are especially vulnerable to degradation by *P. aeruginosa*, but the process is complex, requiring both uptake of these hydrophobic compounds, as well as their subsequent oxidation [10, 12]. *N*-alkane breakdown is known to require specific alkane hydroxylases, rubredoxins and rubredoxin reductases [12], but little else is understood about the additional adaptations that make certain Pseudomonads more efficient alkane degraders than others.

**Fig. 1:**
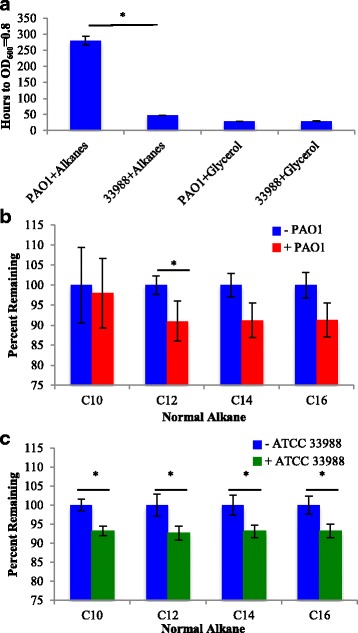
ATCC 33988 degrades *n*-alkanes more rapidly than PAO1. (**a**) Time required for ATCC 33988 and PAO1 cultures to reach OD_600_ = 0.8 when provided with 5% v/v *n*-alkanes or glycerol as the sole carbon source. PAO1 (**b**) and ATCC 33988 (**c**) *n*-alkane degradation profiles at the time of harvesting. All conditions were tested in triplicate. * indicates *p* < 0.05 for data discussed in text

*P. aeruginosa* strain ATCC 33988 (henceforth referred to as ATCC 33988), isolated from a fuel storage tank, shows high sequence identity to PAO1 (average nucleotide identity (ANI) = 99.2) [13], but degrades components of Jet A fuel more rapidly [8]. While Jet A contains a diversity of hydrocarbon species, ATCC 33988 appears to favor specific components for degradation. Amongst the most depleted species are the *n*-alkanes C_10_, C_12_, C_14_, and C_16_ [14]. To determine why ATCC 33988 replicates more efficiently on these hydrocarbons, we compared gene expression in both strains during growth on a 5% (v/v) mixture of *n*-alkanes, composed of equal volumes of the C_8_, C_10_, C_12_, C_14_ and C_16_ species. Additional cultures were grown on 5% glycerol as a control. While ATCC 33988 and PAO1 both grew well on glycerol, reaching OD_600_ = 0.8 after about 24 h, ATCC 33988 grew significantly faster on *n*-alkanes than did PAO1 (2d versus 11d) (Fig. 1a). At the time of harvesting, comprehensive two-dimensional gas chromatography (GCxGC) analysis showed that PAO1 consumed significant amounts of only the C_12_ species while ATCC 33988 significantly depleted C_10_, C_12_, C_14_ and C_16_ (Fig. 1b and c).

**Fig. 2:**
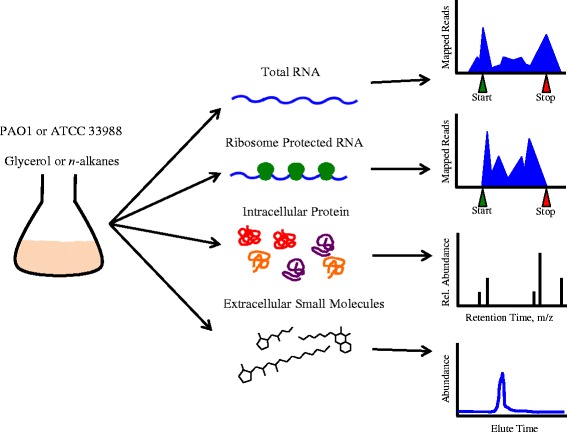
Experimental schematic. Minimal media was inoculated with two different strains of *Pseudomonas aeruginosa* at OD_600_ = 0.02. Glycerol or a mixture of *n*-alkanes were provided as the sole carbon source. Cultures were shaken, and at OD_600_ = 0.8, harvested for total RNA (RNA-seq and microarray), ribosome footprints, intracellular protein and extracellular small molecules

At OD_600_ = 0.8, triplicate samples from each culture condition were harvested and processed for total RNA, ribosome footprints, intracellular protein and extracellular small molecules (Fig. 2). Before any further analysis, both genomes were re-annotated using the RAST (Rapid Annotation using Subsystem Technology) server [15], the Pseudomonas database (PsDB) [16] and the Protein Sequence Annotation Tool [17] (Additional file 1: Tables S1–S4). The Pae_G1a Affymetrix microarray, designed against the PAO1 genome sequence [18], was used to supplement RNA-seq data when ATCC 33988 showed high sequence identity to the cDNA probes (Additional file 2: Tables S5–S8, See Methods for details). Gene expression profiles, as well as translation efficiency values, were generated and compared across each carbon source; PAO1+alkanes vs. ATCC 33988+alkanes (abbreviated as PA vs. 3A), PAO1+glycerol vs. ATCC 33988+glycerol (PG vs. 3G), and across each strain; PAO1+alkanes vs. PAO1+glycerol (PA vs. PG), ATCC 33988+alkanes vs. ATCC 33988+glycerol (3A vs. 3G). Within each comparison, genes whose expression changed by >2-fold, and whose *p*-values were <0.05, were considered “hits” (Additional files 3, 4, 5 and 6: Tables S9–S24).

**Fig. 3:**
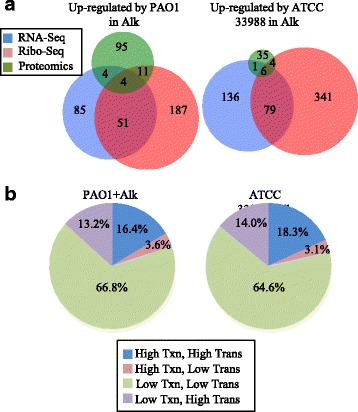
ATCC 33988 and PAO1 induce divergent gene expression profiles when grown on *n*-alkanes. (**a**) Venn diagrams represent the number of *Pseudomonas aeruginosa* genes that are >2-fold increased in one strain when compared to the other during growth in *n*-alkanes (*p* < 0.05). *Blue circle* = RNA-seq, *pink circle* = ribosome footprinting, *green circle* = proteomics. (**b**) Percentages of total analyzed genes that fall within each of four genomic expression categories. Genes are assigned to one of four categories based on whether they appear in the ‘high’ value or ‘low’ value component of the RNA-seq (Transcription/Txn) or ribosome footprint (Translation/Trans) results. See Methods for further explanation of categorization strategy

Both PAO1 and ATCC 33988 induced characteristic expression profiles when exposed to *n*-alkanes (Fig. 3a) and glycerol (Additional file 7: Figure S1A–C). There was substantial overlap in genes induced at the total RNA and ribosome footprint levels, suggesting that when a gene was transcriptionally induced, it was often translationally induced as well. This relationship is mirrored in the sorting of genes into either ‘high’ or ‘low’ transcription/translation groups based on an unsupervised statistical clustering analysis (Fig. 3b and Additional file 7: Figure S1D; for explanation of clustering strategy, see Methods). Most genes are sorted into the same group for transcription and translation (high-high or low-low); it was far more rare for a gene to be classified as highly transcribed and lowly translated or vice versa.

**Fig. 4:**
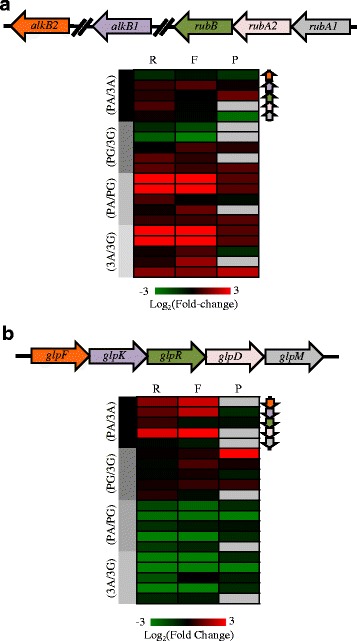
Genes known to be involved in *n*-alkane and glycerol metabolism are induced during growth in their respective carbon sources. Heat maps representing the fold-change in total RNA (1^st^ column, labeled ‘R’), ribosome footprints (2^nd^ column, ‘F’) and protein (3^rd^ column, ‘P’) of genes known to be involved in (**a**) *n*-alkane degradation and (**b**) glycerol degradation. All expression values are log2-transformed. If a protein is not detected in any sample, the corresponding box is labeled *grey*. If a gene is not present in ATCC 33988, the log_2_(fold-change) is the value 1

Aerobic catabolism of *n*-alkanes by *P. aeruginosa* is thought to begin with either a terminal or sub-terminal oxidation step [19]. These reactions require the alkane hydroxylases *alkB1* (PA2574) and *alkB2* (PA1525), the rubredoxins *rubA1* (PA5351) and *rubA2* (PA5350) and the rubredoxin reductase *rubB* (PA5349) [8, 11, 12]. PAO1 and ATCC 33988 encode identical versions of *alkB1/2* and *rubA1/2*, and highly similar versions of *rubB* (>99% sequence identity). While the *rubAB* operon was expressed relatively equally under all conditions, as has been observed previously [20], both alkane hydroxylases were highly induced at multiple levels during growth in alkanes (Fig. 4a). Similarly, when grown in glycerol, both strains saw induction genes of the *glp* operon, which is required for glycerol import and catabolism [21] (Fig. 4b).

**Fig. 5:**
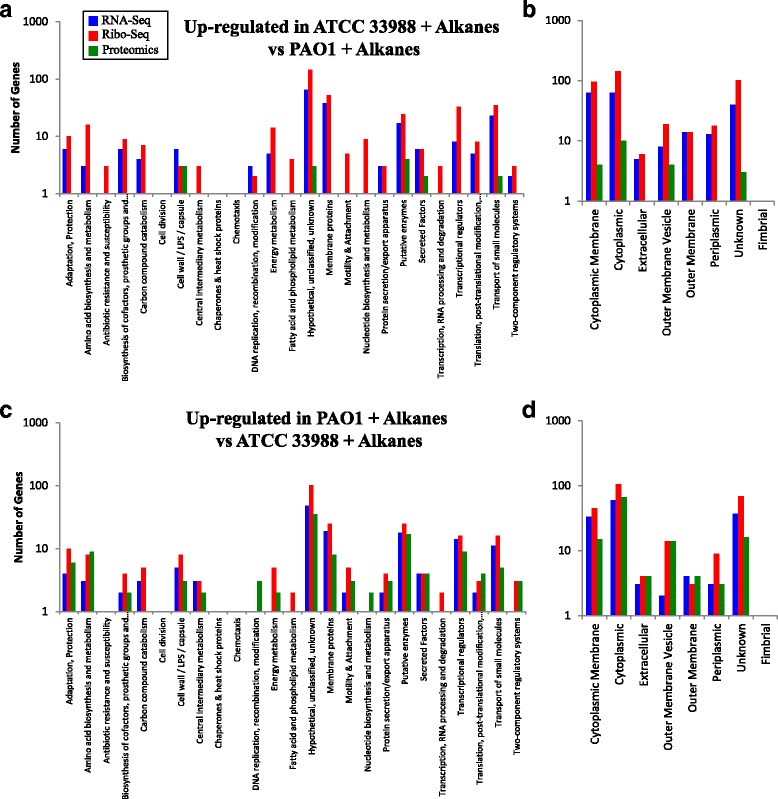
Functional annotation and localization of genes induced by *P. aeruginosa* strains grown in *n*-alkanes. Functional annotations (**a**) and cellular localization (**b**) of genes up-regulated in ATCC 33988 vs PAO1 during growth in *n*-alkanes. (**c** and **d**) Same as above for those genes up-regulated by PAO1 vs ATCC 33988 during growth in *n*-alkanes

Localization and functional analysis underlined the diverse nature of genes induced by each strain in each carbon source (Fig. 5). However, the most highly represented subset of genes induced by ATCC 33988+alkanes vs PAO1+alkanes was those annotated as hypothetical or with no known function, despite using multiple annotation tools (see above). Similar results were previously seen when total RNA from ATCC 33988 grown in Jet A fuel was compared to RNA from ATCC 33988 grown in glycerol [8], emphasizing the need for continued annotation efforts for both *P. aeruginosa* genomes.

To determine how ATCC 33988 replicated so efficiently on *n*-alkanes, we initially focused our analysis on genes differentially expressed by ATCC 33988 versus PAO1 during growth on the hydrocarbon mixture (Fig. 3a). However, as gene expression was measured at a single time point, fold-change values could not distinguish between induction of a gene in 3A vs. PA and repression in PA vs. PG. To differentiate between these two scenarios, we examined fold-changes for each gene in a single strain in both carbon sources (the ratio of PA/PG and 3A/3G). Using this technique, we could predict whether a gene was induced by ATCC 33988 in *n*-alkanes, or if it was instead repressed by PAO1 in *n*-alkanes. The latter was the case for the *oprB* operon, which is involved in carbohydrate transport (PA3186-PA3190) [22] (Additional file 8: Figure S2A). Additionally, by examining the fold-change of a gene between strains in glycerol (PG/3G), we could determine if a gene was induced by one strain compared to the other regardless of the carbon source. This was the case with the toxin-producing *amb* operon (PA2302-PA2306), which is expressed more highly in PAO1 in all conditions tested [23] (Additional file 8: Figure S2B). When analyzed simultaneously, these pair-wise comparisons identified alkane-induced genes in both strains as well as genes differentially expressed regardless of carbon source.

### Rhamnolipid species do not account for the faster *n*-alkane degradation by ATCC 33988

**Fig. 6:**
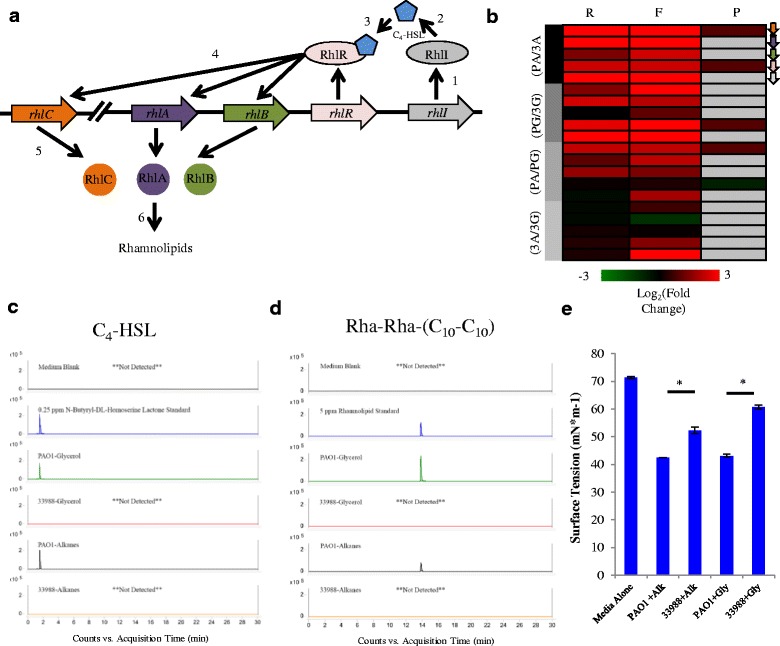
ATCC 33988 does not use rhamnolipids to improve *n*-alkane degradation. (**a**) Rhamnolipid synthesis pathway: RhlI produces the autoinducer N-butyryl-DL-homoserine lactone (C_4_-HSL.) When levels of C_4_-HSL reach a threshold limit, they activate the transcription factor RhlR. RhlR increases transcription of the rhamnolipid biosynthetic enzymes RhlA, RhlB and RhlC, which produce rhamnolipids. (**b**) Heat map representing the fold-change in total RNA (column R), ribosome footprints (column F) and protein (column P) of genes in the rhamnolipid synthesis pathway. All expression values are log_2_-transformed. Undetected genes are shaded grey. If a gene is not present in ATCC 33988, the log_2_(fold-change) is given the value 1. LC-MS traces of the autoinducer molecule C_4_-HSL (**c**) and a sample di-rhamnolipid (**d**) in the extracellular media. **e** Surface tension of each culture. * indicates *p* < 0.05 for data discussed in the text

Due to their low solubility in aqueous conditions, a major limiting step in hydrocarbon degradation by Pseudomonads is bringing the carbon source in contact with the bacteria. Two major strategies have been described to solve this problem; “presolubilization” of hydrocarbons by emulsification, and increasing direct cellular adhesion to the hydrocarbon [24]. *Pseudomonas aeruginosa* strains have the capacity to produce and secrete dozens of extracellular molecules, including the amphipathic bioemulsifiers known as rhamnolipids [25]. These compounds can increase the solubility of alkanes in culture media, thus improving their bioavailability [26, 27]. To determine whether ATCC 33988 increased rhamnolipid production to improve growth on *n*-alkanes, we examined gene expression from the *rhl* rhamnolipid biosynthetic operon (Fig. 6a), and measured small molecule production by accurate mass liquid chromatography-mass spectrometry (LC-MS). In *P. aeruginosa*, rhamnolipid production begins with the expression of RhlI (PA3476), which synthesizes the quorum sensing autoinducer N-butyryl-DL-homoserine lactone (C_4_-HSL) [28]. When levels of C_4_-HSL reach a threshold concentration, it interacts with the transcriptional regulator RhlR (PA3477) to activate transcription of the rhamnolipid biosynthetic genes *rhlA* (PA3479)*, rhlB* (PA3478) and *rhlC* (PA1130). Surprisingly, every component of this core pathway was induced by PAO1 (the slower degrader) when compared to ATCC 33988 (the faster degrader), regardless of carbon source (Fig. 6b). This trend held true at the total RNA, microarray, ribosome footprint and protein (when detected) levels. Consistent with our gene expression findings, we detected C_4_-HSL in the spent liquid medium (SLM) of PAO1 cultures, but not with ATCC 33988 (Fig. 6c). This was also true for rhamnolipid production; PAO1 produced several rhamnolipid species in the SLM: the di-rhamno-di-lipidic congeners Rha-Rha-(C_10_–C_10_), Rha-Rha-(C_10_–C_12_) and Rha-Rha-(C_10_–C_12:1_), as well as the mono-rhamno-di-lipidic congener Rha-(C_10_–C_10_). Extracted ion chromatograms (EIC) of the most abundant rhamnolipid Rha-Rha-(C_10_–C_10_) are shown in Fig. 6d. No rhamnolipid production was detected for ATCC 33988.

In addition to increasing alkane solubility, rhamnolipids also decrease surface tension at the media/air boundary [29]. We observed that surface tension decreased significantly in PAO1+glycerol and PAO1+alkane samples compared to media alone, while a more modest decrease was seen in ATCC 33988 only after growth in alkanes (Fig. 6e).

As mentioned above, cells can also increase contact with hydrocarbons by improving the direct adhesion of these compounds to the cell surface. This often involves an increase in cell surface hydrophobicity, which can be measured by the bacterial adhesion to hydrocarbons (BATH) assay [30]. We found that PAO1 is more hydrophobic than ATCC 33988, regardless of growth in glycerol or *n*-alkanes (Additional file 9: Figure S3).

### ATCC 33988 does not encode the *lasI*/*lasR* components of the quorum sensing response

**Fig. 7:**
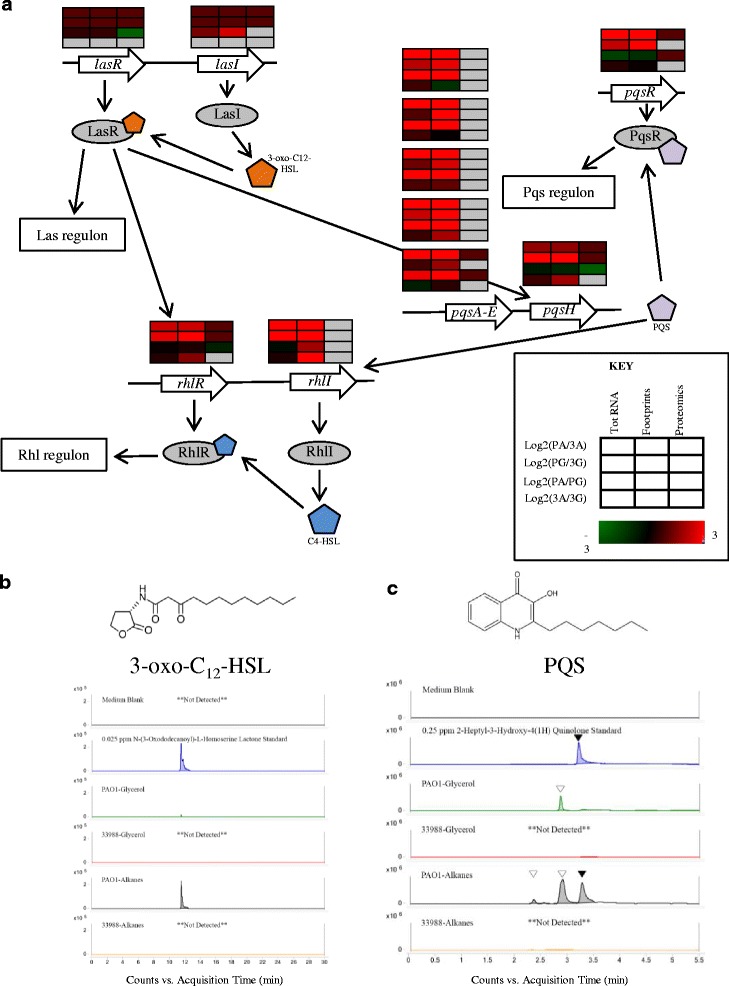
ATCC 33988 shows muted expression from the QS network. (**a**) Schematic showing QS autoinducer synthesis genes and cognate receptors for in *Pseudomonas aeruginosa* as discussed in text. LC-MS traces of the autoinducer molecules (**b**) 3-oxo-C_12_-HSL and (**c**) PQS in the extracellular media. *Triangles* represent putative PQS isomers with different retention times but the same exact mass as the PQS standard

Rhamnolipids are produced as part of the quorum sensing (QS) response in *P. aeruginosa*, a complex, interregulated system that controls the transcription of certain genes based on cell density [31]. Many genes in the QS regulon, in addition to the *rhl* operon, have been predicted to play a role in alkane degradation, including those involved in the formation of biofilms, iron acquisition or efflux pumps [8]. Along with *rhl*, there are two other well-characterized branches of the QS system, *las* and *pqs*, each with their own autoinducer molecules. Just as with C_4_-HSL, at a critical concentration, the Las and Pqs autoinducers bind to cognate transcription factors, which induce or repress a downstream regulon (Fig. 7a). In the *las* system, LasI (PA1432) produces the signaling molecule N-3-oxododecanoyl-L-homoserine lactone (3-oxo-C_12_-HSL), which binds to LasR. In the *pqs* system, quinolones are used as autoinducers. Specifically, pqsABCDE (PA0996-0998 and PA1000-1001) and pqsH (PA2587) synthesize 2-heptyl-3-hydroxy-4(1H)-quinolone (PQS), which binds to PqsR (PA1003).

**Fig. 8:**
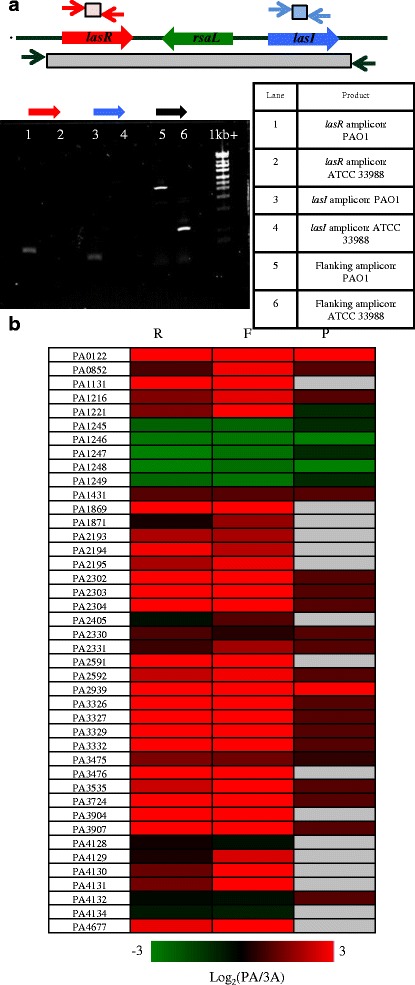
ATCC 33988 does not encode *lasI*/*lasR*. (**a**) Genome organization of the *lasR*-*lasI* fragment of PAO1. To evaluate the presence or absense of the *lasI*/*lasR* genes in ATCC 33988, three sets of PCR primers were designed. The *red* and *blue primer* pairs amplify regions within the *lasR* and *lasI* genes, respectively. Expected PCR product sizes were 295 bp (*lasR*) and 204 bp (*lasI*). The third primer pair (*black*) anneals to the region flanking the *lasR*/*lasI* region, which is sequence-conserved in both strains. When both *lasR* and *lasI* were present in this region, the expected PCR product size was 2.4 kbp. All PCR products were run on a 1.2% agarose gel. (**b**) Heat map representing the fold-change in gene expression between PAO1 in alkanes and ATCC 33988 in alkanes. All expression values are log_2_-transformed. If a gene is not detected in any sample, the corresponding box is shaded *grey*. If a gene is not present in ATCC 33988, the log_2_(fold-change) value is assigned to 1. R = total RNA, F = ribosome footprints, P = proteomics

Similarly to the *rhl* operon, total RNA, ribosome footprint and protein levels of the *las* and *pqs* regulator genes were induced in PAO1+alkanes versus ATCC 33988+alkanes (Fig. 7a). In support of this result, the two corresponding autoinducers, 3-oxo-C_12_-HSL and PQS, were present at higher levels in PAO1 cultures as determined by LC-MS analysis, and in particular in PAO1+alkane cultures (Fig. 7b and c). The lack of 3-oxo-C_12_-HSL in ATCC 33988 cultures was especially telling, as it is the *las* system that is primarily responsible for the initial induction of both the *rhl* and *pqs* systems under standard laboratory conditions [32]. Due to the decreased levels of QS autoinducers in ATCC 33988 SLM, we hypothesized that this strain may lack the *lasR* and *lasI* genes, resulting in a muted QS response. Indeed, PCR amplification of the sequence-conserved regions surrounding *lasR* and *lasI* showed that ATCC 33988 does not encode either of these genes (Fig. 8a). These results suggest that ATCC 33988 does not use the QS response in the same fashion as PAO1, and that canonical QS induction is not responsible for ATCC 33988’s increased *n*-alkane degradation rates.

In different strain backgrounds and environmental conditions, the response to LasI and 3-oxo-C_12_-HSL production varies. However, there is a set of “core quorum-controlled genes” thought to be induced by *P. aeruginosa* acyl-serine lactones (the collective term for autoinducers 3-oxo-C_12_-HSL and BHL) regardless of ecological niche [33]. Total RNA, footprint and protein levels of nearly all of these genes were induced in PAO1+alkanes versus ATCC 33988+alkanes (Fig. 8b). The exception to this phenotype was the set of genes PA1245-1249, which were all induced in ATCC 33988+alkanes.

### Multi-omics identifies operons induced by ATCC 33988 during growth on *n*-alkanes

**Fig. 9:**
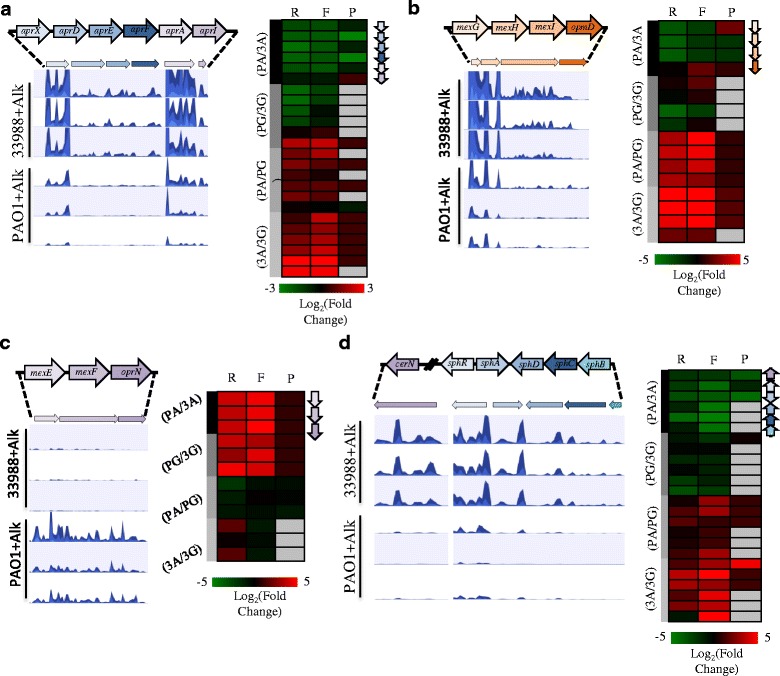
Gene expression from the *apr*, *mexGHI-opmD* and *sphR* operons were induced by ATCC 33988 growth in *n*-alkanes. Each panel portrays the operon organization, ribosome footprint traces and gene expression profiles (R = total RNA, F = ribosome footprints, P = protein) for the indicated set of genes (**a**) *apr* operon, (**b**) *mexGHI*-*opmD* operon, (**c**) *mexEF-oprN* operon, and (**d**) *sphR* regulon. For ribosome footprinting traces, each row represents one biological replicate

Five genes of the *apr* operon (PA1245-1249) were the sole representatives from the set of core QS-regulated genes that were induced by ATCC 33988+alkanes versus PAO1+alkanes (Figs. 8b and 9a). This operon encodes the machinery for the production and secretion of the proteases AprA and AprX [34]. In addition to being induced by the QS apparatus, *aprA* is positively regulated by the bi-stable transcription regulator BexR (PA2432). Interestingly, ectopic expression of BexR has previously been shown to increase *mexGHI*-*opmD* (PA4205-4208) expression and decrease *mexEF*-*oprN* (PA2393-2495) expression [35]. Both of these phenotypes were also observed when comparing growth of ATCC 33988 and PAO1 in alkanes (Fig. 9b and c).

By expanding our examination to include non QS-sensitive genes, we identified an additional sets of genes more heavily induced by ATCC 33988 than PAO1 in *n*-alkanes. The SphR regulon includes the five consecutive genes PA5324-5328, as well as the upstream neutral ceramidase (PA0845). Each of these genes was highly induced by ATCC 33988 in *n*-alkanes (Fig. 9d). In addition to being induced intracellularly by ATCC 33988, the CerN protein was also present at high levels in the extracellular media of the environmental isolate (data not shown). These genes are all transcriptionally induced by sphingosine [36, 37], but their roles in alkane degradation pathways are not known. No sphingosine was present in the extracellular media of any cultures.

## Discussion

In the present study, results from transcriptome, translatome, proteome and small molecule analyses were integrated to better understand gene expression changes that occur during growth in *n*-alkanes by related strains of *Pseudomonas aeruginosa*. These large data sets both confirm and extend previous findings from microarray analyses of these bacteria during growth on petroleum hydrocarbons [8, 14]. *P. aeruginosa* strain ATCC 33988, isolated from a fuel storage tank, has been shown to grow significantly faster on jet fuel than the laboratory strain PAO1 [14], and we found that this increased growth rate is also observed during growth in C_8_–C_16_ *n*-alkanes (Fig. 1). Since these strains show a high level of genome sequence identity, the adaptations that make ATCC 33988 more efficient in *n*-alkane degradation are likely due to differences in the regulation of gene expression. By combining information about the transcription, translation, protein and small molecule profiles of ATCC 33988 and PAO1, we were able to determine where gene expression deviated between the strains. This information led to several testable hypotheses, and has provided possible explanations for why ATCC 33988 performs better when grown on *n*-alkanes.

### Transcription and translation of most *P. aeruginosa* genes are induced or repressed in tandem

One weakness of studies that report only RNA measurements is the poor correlation sometimes seen between mRNA and protein [38, 39]. This weak relationship between transcript and protein levels is likely due to the multiple regulatory steps that occur co- and post-transcriptionally, which makes it difficult to draw accurate conclusions about the cellular environment based solely on microarray or RNA-seq data. For ATCC 33988 and PAO1, however, we found that for many genes, an increase in total RNA values under one condition frequently occurred alongside an increase in ribosome footprint levels (Fig. 3a). For a large percentage of genes (>80%), the mRNA abundance level, when categorized into “high” or “low” bins, matched translation level categorizations (Fig. 3b). This trend was also present, but less robust, when comparing total RNA and footprint levels to protein levels. These results suggest that regulation of gene expression for these two strains of *P. aeruginosa* often begins during transcription, and that when gene transcription is induced, translation is also induced by at least the same, if not greater, magnitude. A prime example of this pattern is seen with *alkB1* and *alkB2*, the two alkane hydroxylases that catalyze the initial oxidation step in alkane degradation [12]. When comparing expression levels of these genes in ATCC 33988+alkanes versus glycerol, transcription was induced by 12-fold (*alkB1*) and 41-fold (*alkB2*), footprints were induced by 43- and 217-fold, and both proteins were highly expressed in alkane cultures while absent in glycerol cultures (Additional files 3, 4 and 6). This trend was maintained in measurements of translation efficiency (amount of translation per transcript), specifically for *alkB2* during growth in alkanes (Additional file 5). Expression of these genes in PAO1 showed a similar pattern.

### Strain ATCC 33988 shows a reduced quorum-sensing response

ATCC 33988 grew much faster than PAO1 on *n*-alkanes, and we expected gene expression profiles to reflect this disparity. Many of the most significant differences between the two strains during growth on *n*-alkanes appeared in genes involved in the cell density-dependent quorum sensing (QS) response. QS plays a complex, important role in the *P. aeruginosa* life cycle, and hundreds of genes have been shown to be responsive to QS signaling molecules under varying conditions [25]. The functions of these genes are diverse, contributing to everything from biofilm formation to virulence. QS is highly regulated, and at least three autoinducer molecules activate cognate transcription factors that act on various sub-populations of the regulon. Our total RNA and ribosome footprint data showed that ATCC 33988 had little to no transcription or translation of any of the major autoinducer biosynthesis genes (*rhlI*, *lasI*, *pqsA-H* and corresponding proteins) or their complementary receptors (*rhlR*, *lasR*, *pqsR*, and corresponding proteins) when compared to PAO1 (Figs. 6 and 7). This observation suggested that ATCC 33988 was unlikely to synthesize any of the QS autoinducers (i.e. 3-oxo-C_12_-HSL, C_4_-HSL and PQS), which was supported by small molecule analysis (Figs. 6 and 7).

Given that only very low levels of autoinducer molecules were present in ATCC 33988 cultures, it was unsurprising that ATCC 33988 did not express many of the “core” QS-responsive genes to the same extent as PAO1 (Fig. 8b). The current view is that while each of the three major branches of the QS network can affect the others, the Las branch sits at the top of the hierarchy [40]. ATCC 33988 showed no expression of LasI or LasR at the transcription, translation or protein levels, contributing to the low expression from the *rhl* and *pqs* operons. As before, results from the initial -omics experiments led to a prediction about ATCC 33988, this time that it may lack the *lasI* and *lasR* genes. Subsequent PCR amplification proved that ATCC 33988 did not encode *lasI* or *lasR* in its genome (Fig. 8a).

A major downstream effect of QS induction by PAO1 versus ATCC 33988 was the increased transcription and translation of the rhamnolipid synthetic genes *rhlA*, *rhlB* and *rhlC* in PAO1. Rhamnolipids can be used to disperse hydrophobic compounds in aqueous environments, and when access to *n*-alkanes is a rate-limiting step in their consumption, increased rhamnolipid secretion could improve *Pseudomonas* growth on *n*-alkanes. Previous work, for example, has shown that exogenous rhamnolipids improve octadecane degradation by *P. aeruginosa* strain ATCC 9027 [29], and other studies have reported similar growth advantages with different hydrocarbon mixtures [41–44]. However, despite the fact that ATCC 33988 grew faster in *n*-alkanes, it had minimal expression from the *rhl* operon, which suggested that it didn’t produce this class of molecule. LC-MS analysis supported this hypothesis. These results show that rhamnolipids are not necessary for all *P. aeruginosa* species during growth on specific hydrocarbons, and they do not contribute to the faster growth of ATCC 33988 on our *n*-alkane mixture.

Even without producing rhamnolipids, ATCC 33988 did show a small decrease in surface tension during growth on *n*-alkanes, suggesting the strain produces an alternative biosurfactant/bioemulsifier. Microbial surfactants can have a variety of structures, including glycolipids (like rhamnolipids), phospholipids, fatty acids and proteins. Both *P. aeruginosa* strains used in this study encode the “protein activator” *pra* (PA4590), which has been shown to possess emulsification activity and improve *P. aeruginosa* growth on hexadecane in the presence and absence of rhamnolipids [11, 45]. Our data showed that transcription and translation of *pra* were induced by ATCC 33988 (and PAO1) during growth in alkanes (Additional files 2, 3, 4 and 5, Additional file 10: Figure S4). It is possible, then, that the smaller decrease in surface tension induced by ATCC 33988 during growth on *n*-alkanes may be due to Pra. Future experiments measuring surface tension and *n*-alkane degradation by ATCC 33988 in the absence of Pra would provide direct clues as to its role in these processes.

### Divergent models for *n*-alkane uptake in strains of *P. aeruginosa*

*N*-alkane degradation requires two separate steps: uptake and catabolism. Our results suggest that PAO1 utilizes two strategies to improve alkane uptake: pre-solubilizing hydrophobic compounds and increasing cell surface hydrophobicity. Pre-solubilization (and decreased surface tension) are likely achieved through the emulsification activity of rhamnolipids and the protein Pra [45], both of which were induced during growth on *n*-alkanes. Increased surface hydrophobicity was also likely due to rhamnolipid production, as they have been shown previously to have this effect on Pseudomonads grown in certain conditions [46, 47].

ATCC 33988 did not produce significant levels of rhamnolipids, which likely explains its higher surface tension and lower surface hydrophobicity measurements. How, then, does ATCC 33988 uptake *n*-alkanes? As mentioned above, ATCC 33988 induced the expression of Pra during growth in *n*-alkanes. Rhamnolipids and Pra, while both emulsifiers, have very different structures, and potentially different environmental niches in which they are most useful. As ATCC 33988 was isolated from a fuel storage tank, and grows more quickly on *n*-alkanes, it is possible that it has evolved to decrease rhamnolipid production because they are inhibitory or unhelpful to its growth on hydrocarbons. In fact, previous studies have found that addition of biosurfactant to environmental isolates of bacteria are equally as likely to have a positive, negative, or neutral effect on the degradation of hydrophobic compounds [48, 49]. Our data suggests that the role of rhamnolipids in uptake of hydrophobic substrates may be more complex than originally thought, and emphasizes the importance of compatibility between surfactant and microbe.

### -Omics data identifies candidate genes potentially responsible for the improved growth and/or survival of ATCC 33988 on *n*-alkanes

While expression from the *rhl* and *las* operons were primarily limited to PAO1, ATCC 33988 did induce some genes during growth on *n*-alkanes when compared to PAO1. This expression pattern makes these genes attractive candidates for further exploration regarding their roles in ATCC 33988’s more efficient growth/survival on our *n*-alkane mixture. One robust example was the *apr* operon (Fig. 8a). The genes *aprX* and *aprA* showed especially large changes in expression as compared to PAO1 in alkanes. Both of these proteins are secreted proteases, and while the substrate of AprX is not known, AprA can cleave several components of the host immune system [50]. As they are found outside of the cell, one or both of these proteases may have access to proteins of the bacteria-produced extracellular polymeric substance (EPS). While far from conclusive, there is evidence of EPS inhibiting adherence of bacteria to hydrocarbons [51], and AprA/X could potentially act to degrade the proteinaceous component. Further determination of AprX substrate specificity will help answer this question.

ATCC 33988 grown in *n*-alkanes also induced expression of the SphR regulon to a higher degree than PAO1 (Fig. 9d). Little is known about the SphR regulon, other than the fact that SphR binds, and is responsive to, exogenous sphingosine, dihydrosphingosine and phytosphingosine [36, 37]. Interestingly, sphingosine is a component of human lung surfactants, and while ATCC 33988 does not produce rhamnolipids, it is possible that it synthesizes a yet unidentified surfactant species in the presence of *n*-alkanes, and that this compound may be structurally similar to sphingosine, and more compatible with ATCC 33988 than rhamnolipids.

Once internalized, alkanes are catabolized through the action of alkane monooxygenases *alkB1*/*B2*, rubredoxins *rubA1*/*A2* and rubredoxin reductase *rubB*. Both ATCC 33988 and PAO1 encode these enzymes, and the monooxygenases, in particular, are highly induced by *n*-alkanes. Both the sub-terminal and terminal oxidation strategies for alkane degradation involve the downstream enzymatic activity of an alcohol dehydrogenase (ADH) to eventually convert the alkanes into fatty acids that can be degraded by β-oxidation. PAO1 and ATCC 33988 encode more than a dozen known and putative ADH enzymes, but of particular interest is PA2275, which is induced by ATCC 33988+alkanes at both the transcriptional and translational levels when compared to PAO1+alkanes. More work needs to be done on the substrate specificity of PA2275, but it is possible that ATCC 33988 uses this enzyme to more efficiently catabolize alcohols formed during the degradation of *n*-alkanes.

It also must be mentioned that this analysis identified several hundred genes found only in one strain or the other. More than half of these genes were not functionally characterized by any annotation tool used in this study, and none were annotated as alkane hydroxylases, rubredoxins or rubredoxin reductases. As such, their role in the metabolism of *n*-alkanes remains to be addressed.

### The role of multi-omics experiments in the field of systems biology

In this study, we show the power of integrating multiple –omics data sets towards a better understanding a complex biological process: *n*-alkane degradation in *Pseudomonas aeruginosa*. By collecting expression data from RNA-seq, microarray, ribosome footprinting, proteomic and small molecule mass spectrometry experiments, we were able to not only confirm previously observed phenotypes (i.e. the role of *alkB1* and *alkB2*), but were able to generate and test new hypotheses regarding hydrocarbon consumption by bacteria. This type of integrative system biology is especially useful when dealing with draft, or incompletely annotated, genomes, as RNA-seq can provide information about 3′ and 5′ untranslated regions and transcription start sites, while ribosome footprinting can elucidate frameshifts, alternative start sites and nested genes. Our data allowed us to update gene annotations based on expression under certain conditions, and also identified dozens of genes that were unique to one strain or another, which could play a role in adapting to a hydrocarbon environment. While typically less sensitive than sequencing-based tools, proteomics can give information about localization and post-translational modifications, and to complete the process, the reagents and products of enzymatic reactions can be quantified by targeting specific small molecules for quantification (Table 1).

**Table 1:** Strengths and weaknesses of -omic techniques

Technique	Advantages	Disadvantages
RNA-seq	- Established protocols - Many commercially available kits - Captures both translated and non-translated regions of RNA	- mRNA abundance does not always correlate with protein abundance - Expense and turn around time
Ribosome Footprinting (Ribo-seq)	- Global view of all translated mRNA - Nucleotide/codon-level resolution - Identifies unique genomic sites: frameshifts, splice isoforms, ribosome pause sites	- Technically more difficult - Time-consuming post-sequencing analysis - Expense
Proteomic LC-MS	- Represents net result of transcriptional and translational regulation - Deconvolutes complex protein mixtures - Simple to gather intra- and extracellular protein data	- Typically identifies fewer genes than nucleic acid-based tools - No individual method to separate all proteins
Small Molecule LC-MS	- Measures end product of enzymatic activity - Standards can be used for simple quantitation	- Prior knowledge of elution time and exact mass helpful - Different separation techniques for classes of molecules

*LC-MS* liquid chromatography-mass spectrometry

Moving forward, tools that integrate -omics data sets must continue to improve and become more user-friendly. As protocols advance and allow for higher throughput and higher resolution data gathering, the future of the field lies in the ability to both vertically and horizontally integrate this new information.

## Conclusions

Despite the advancements that have been made in high-throughput technologies, integration of these data sets, and elucidation of biological meaning remains a challenge. In this study, a unique set of -omics tools were combined to generate a holistic picture of normal alkane degradation by two strains of *Pseudomonas aeruginosa*. Each strain utilizes a different set of genes for growth on alkanes, and the environmental isolate ATCC 33988 does so without the full quorum sensing response, and with little-to-no production of rhamnolipids. Several operons are uniquely induced by ATCC 33988, and these genes represent the most appealing choices for further mechanistic examination. This genome-wide, systems-based strategy not only uncovers important layers of regulation used by each strain during growth on alkanes, but additionally generates specific, testable hypotheses.

## Methods

### Bacterial strains and growth conditions

The laboratory strain *Pseudomonas aeruginosa* PAO1 (taxonomy ID 208964.1) was used as the wild-type control. *P. aeruginosa* ATCC 33988 (QMB 1592) was isolated from a fuel storage tank in Ponca City, OK [13]. Single colonies of both strains were grown in LB overnight at 28 °C. Cells were pelleted (5000 rpm, 7–10 min) and washed three times with fresh M9 minimal media (90 mM Na_2_HPO_4_*7H_2_O, 22 mM KH_2_PO_4_, 8.5 mM NaCl, 18.6 mM NH_4_Cl, 2 mM MgSO_4_, 0.1 mM CaCl_2_ and 0.02 mM FeSO_4_) before being diluted to an OD_600_ = 0.02 in 250 mL of M9. Carbon sources (glycerol or an equal mixture of C_8_, C_10_, C_12_, C_14_, and C_16_ normal alkanes) were added to a final concentration of 5% (vol/vol). Cultures were grown with vigorous shaking in a baffled flask with loose lids to an OD_600_ of 0.8. During growth on alkanes, both strains produced modest amounts of hydrophobic extracellular material that artificially increased OD_600_ measurements. To ensure that all samples were harvested at the same cell density, aliquots of each culture were pelleted and resuspended in fresh minimal media before taking absorbance measurements. At the time of harvesting, small aliquots (several mL) of each culture were removed for alkane degradation, microarray, RNA-seq and proteomic analysis.

#### Alkane degradation

Unconsumed alkanes were extracted and analyzed by GCxGC as in [8]. All degradation values were normalized to cultures incubated without bacteria. Accurate determination of C_8_ consumption was complicated by its increased volatility as compared to heavier alkane species, so it was not included in our analysis.

#### Total RNA isolation and microarray analysis

For RNA-seq and microarray analysis, total RNA was isolated using TRIzol MAX Bacterial RNA isolation kit (Ambion, Foster City, CA, USA), and DNA contamination was removed using the TURBO DNA-free kit (Invitrogen, Carlsbad, CA, USA). For microarray analysis, cDNA was synthesized as in [8] and applied to the Affymetrix gene-chip of *P. aeruginosa* PAO1 (Pae_G1a) (Affymetrix, Santa Clara, CA, USA). Arrays were washed and stained as described in the Technical Manual. Initial data analyses were performed as in [8], using the Affymetrix Microarray Analysis Suite. As the microarray panel was designed using the PAO1 sequence, genes were only considered for further analyses if the ATCC 33988 sequence showed high hybridization potential (90% sequence alignment and 85% sequence similarity within that alignment) for ≥11 of the 13 probes targeting that gene. Of the 1554 genes in the microarray, 137 (8.8%) did not meet this threshold and were discarded.

### Ribosome profiling

Ribosome footprinting, and the subsequent analysis was performed as in [52], with slight modifications. Briefly, cultures that reached OD_600_ = 0.8 were treated with chloramphenicol (100 ug/mL) for 2 min before being mixed with an equal volume of chloramphenicol-ice. Samples were centrifuged (4500 × *g*, 10 min, 4 °C) and pellets were washed with 5 mL cold resuspension buffer (20 mM Tris, pH 8, 10 mM MgCl2, 100 mM NH4Cl, 1 mM chloramphenicol). Resuspended cells were pelleted again (3000 × *g*, 5 min, 4 °C) and suspended in polysome lysis buffer (20 mM Tris, pH 8, 10 mM MgCl2, 100 mM NH4Cl, 0.4% Triton X-100, 0.1% NP40, 100 U/mL DNase I, 0.5 U/uL Superase•In (Ambion, Foster City, CA, USA), 1 mM chloramphenicol) before being flash frozen in liquid nitrogen.

Cells were lysed by cryogenic pulverization using a CryoMill (Retsch MM301, Newton, PA, USA) for 5 cycles at 15Hz (3 min per cycle), with a re-chilling period between each cycle. Pulverized cells were thawed and incubated with DNase I (NEB, Ipswich, MA, USA) for 10 min on ice. Supernatants were clarified by centrifugation (20,000 × *g*, 10 min, 4 °C). One milligram aliquots were flash frozen in liquid nitrogen. One aliquot was thawed and treated with 1000 units of MNase (NEB) and Superase•In, and a control aliquot was treated with Superase•In alone. All samples were incubated for 75 min at room temperature. MNase activity was quenched with 6 mM EDTA.

Sucrose solutions of 10 and 50% were prepared in polysome gradient buffer (10 mM MgCl2, 100 mM NH4Cl, 20 mM Tris, pH8, 1 mM chloramphenicol, 20 U/mL Superase•In) and gradients were formed using a BioComp Gradient Master (BioComp Instruments, Fredericton, Canada). Digested and undigested footprint samples were loaded onto gradients and spun in a SW41 rotor (217,000 × *g*, 2.5 h, 4 °C). Gradients were fractionated at 0.3 mm/s while A_260_ was continuously monitored. Fractions representing monosome peaks were collected and flash-frozen. Monosome fractions were denatured in a total volume of 700 uL of cleavage buffer (50 mM Tris, pH 7, 200 mM NaCl, 10 mM MgCl2, 1 mM chloramphenicol) and RNA was extracted with hot acid phenol.

Intact total RNA samples (100 to 500 ng) were fragmented in 3.3x T4 Polynucleotide Kinase Reaction Buffer for 8 min at 94 °C for 8 min to an average size of approximately 30 nucleotides. Fragmented total RNA samples and purified Ribosome protected fragments were end repaired with T4 Polynucleotide Kinase (NEB) and purified with a Zymo Research RNA Clean and Concentrator-5 Kit (Zymo Research Corporation, Irvine, CA, USA). RNA sequencing libraries were prepared from the end repaired RNA as described using the NEBNext Multiplex Small RNA Library Prep Set for Illumina (Illumina, San Diego, CA, USA). Following PCR amplification, the library was size selected to enrich for adapter-ligated molecules with an average insert size of 30 bp. Size selection was performed on a Sage Science 3% agarose, dye free, Pippin Prep Gel Cassette with internal markers (Sage Science, Beverly, MA, USA) using the following parameters: BP Start = 105 bp, BP End = 170 bp. Size selected libraries were qualified on an Agilent 2200 TapeStation High Sensitivity D1000 ScreenTape assay (Agilent Technologies, Santa Clara, CA, USA) and the molarity of adapter-modified molecules was defined by quantitative PCR using the Kapa Library Quant Kit (Kapa Biosystems, Wilmington, MA, USA). The molarity of individual libraries were normalized to 5 nM and equal volumes were pooled in preparation for Illumina sequence analysis.

Sequencing libraries (25 pM) were chemically denatured and applied to an Illumina HiSeq v4 single read flow cell using and Illumina cBot. Hybridized molecules were clonally amplified and annealed to sequencing primers with reagents from an Illumina HiSeq Cluster Kit v4-cBOT (CS-401-4001). Following transfer of the flowcell to an Illumina HiSeq 2500 instrument (HCSv2.2.38 and RTA v1.18.61), a 50 cycle single-read sequence run was performed using HiSeq SBS v4 sequencing reagents.

The initial evaluation of the base quality was performed with FastQC v.0.11.4 (Babraham Bioinformatics, United Kingdom) [53]. The reads were trimmed and filtered for quality, adapters removed, and minimum read length set to seven nucleotides using CLC Genomic Workbench v9.0 (Qiagen, Germantown, MD, USA). Bowtie v1.1.1 was used to screen all trimmed fastq files to exclude reads derived from ribosomal contamination using an index of all *P. aeruginosa* PA01 rRNA and tRNAs [54]. Within the CLC Genomics Workbench, trimmed rRNA depleted reads were mapped using the RNA-seq analysis package (mismatch cost: 2, insertion cost: 3, deletion cost: three length fraction: .90, and similarity fraction: 0.85; with maximum number of hits for a read set to 10) against either *P. aeruginosa* PA01 complete genome or the *P. aeruginosa* ATCC 33988 draft genome. The reference genome, *P. aeruginosa* PA01 downloaded from NCBI (NC_002516), and the draft assembly for *P. aeruginosa* ATCC 33988 (GenBank JPQQ00000000) were submitted for annotation to the RAST annotation server [15].

#### Exponential-gamma mixture models for unsupervised clustering of high-throughput sequencing data

To assess differences in high-throughput genomic observations (e.g. reads per kilobase per million reads (RPKM) values), we took a novel unsupervised statistical approach based on two observations: 1) RPKMs are positively valued and 2) genes in each strain/condition can be coarsely categorized as having either *high* or *low* values for that class of observation. We cluster genomic observations for all genes using a mixture of gamma distributions, an approach yet to be applied to these data to the best of our knowledge. Without specifying any thresholds, we separate the *high* and *low* clusters by constraining the *low* component (where values are near zero) to be an exponential distribution, a special case of the gamma distribution. The *high* component is a gamma distribution. More formally:

$$ {X}_{i,1},{X}_{i,2},{X}_{i,3}\sim {\uppi \mathrm{Exp}}_{low}\left(\uplambda \right)+\left(1-\uppi \right){\mathrm{Gamma}}_{high}\left(\alpha, \beta \right) $$

Here, X_i,1_, X_i,2_, and X_i,3_ are the three replicate genomic measurements for gene *i*. π is the component weight (or proportion of all genes) in a given strain-nutrient condition that have a *low* value. 1/λ is the mean of the low-valued genomic measurements and α/β is the mean of the high-valued measurements. The replicate measurements are assumed independently distributed from one another, and each gene is categorized as belonging to either the *low* or *high* value component. We fit this model separately to total RNA (RPKM), ribosome occupancy (RPKM) and proteomic (NSAF, or normalized spectral abundance factor) observations of more than 5200 genes from PAO1 and 33988 in alkanes.

To estimate parameters of the model, we used the software package JAGS (http://mcmc-jags.sourceforge.net/) in conjunction with the R statistical programming environment (https://cran.r-project.org/). To carry out Bayesian inference, we specified priors on model parameters. The prior distributions were based on empirical observations when available and were made as weak as possible otherwise so as not to overly bias estimation. The prior distributions were as follows:

- λ ~ Gamma (5.317999, 11.546939)
- *α* ~ Unif (1e-08, 10^4^ ∙ ($$ \widehat{\alpha} $$ − 1e-08)
- *β* ~ Unif (1e-08, 10^4^ ∙ ($$ \widehat{\beta} $$ − 1e-08)
- *π* ~ Beta (1, 1)

### Proteomic mass spectrometry

Intracellular proteins were harvested using the ProteaPrep Anionic Cell Lysis Kit (Protea Biosciences, Morgantown, WV, USA) following the manufacturer’s instructions. Cell lysates were treated with Benzonase and the acid-labile surfactant was degraded using formic acid. Proteins were precipitated using trichloroacetic acid (TCA). Extracellular proteins were TCA precipitated after an initial centrifugation step (12,000 × *g*, 10 min, 4 °C) to pellet cells.

Precipitated proteins were resuspended in 40% methanol, 100 mM NH_4_HCO_3_ (pH 8.5) and 5 mM TCEP before being incubated at room temperature for 20 min. Iodoacetamide was added to a final concentration of 10 mM and incubated at room temperature for 15 min in the dark. CaCl_2_ was added to 1 mM, trypsin was added to 0.5 ug/uL and the samples was incubated at 37 °C overnight in the dark. Formic acid was added to the digested peptide mixture to a final concentration of 5%, and samples were subjected to MudPIT analysis [55] at the University of California at Berkeley QB3 Proteomics/Mass Spectrometry Laboratory.

### Liquid chromatography-electrospray ionization-mass spectrometry (LC-ESI-MS)

Rhamnolipids and QS molecules were detected in spent liquid medium using an Agilent 6550 Accurate-Mass TOF LC-MS system. A reversed-phase Zorbax Eclipse Plus C18 (RRHD) column (2.1 × 100 mm, and 1.8 μm particle size) was used at 30 °C in an Agilent 1290 HPLC Separation Module connected to an Agilent 6550 iFunnel Q-TOF LC-MS system equipped with a an Agilent Jet Stream II dual sprayer ESI source. Mobile phases consisted of water-formic acid (99.9%:0.1%) (solvent A) and 100% acetonitrile (solvent B). The following solvent composition program was used: isocratic 0.5 min of 20% of solvent B, gradient for 19.5 min until 95% solvent B, isocratic 10 min with 95% solvent B, then an equilibration time of 5 min (post time). The flow rate was kept constant at 0.3 mL/min, the injection volume was 10 μl (with needle wash), and the samples were maintained at 4 °C inside the autosampler. The LC-MS instrument was operated in positive ion electrospray mode with an acquisition range of 115–1700 amu with a scan rate of 3 spectra/s. The source was kept at 225 °C with a gas flow of 17 l/min, and a sheath gas temperature of 380 °C and sheath gas flow of 12 l/min. The VCap was set at 3500, the nozzle voltage at 500 V, the fragmentor at 150, Skimmer1 to 0, and octopole RF peak to 750. Data acquisition was performed using Agilent MassHunter LC-MS Data Acquisition Software (version B.05.01, build 5.01.5125.2). Data analysis was performed using Agilent MassHunter Qualitative Analysis Software (version B.06.00, build 6.0.633.0).

#### Determination of cell surface hydrophobicity

Cell surface hydrophobicity was measured using the bacterial adhesion to hydrocarbon (BATH) assay as described in [30]. Briefly, cells were grown as in the ribosome profiling experiment. When OD_600_ = 0.8, cells were washed five times and resuspended in M9 media at an OD_600_ of 1.0. Five hundred microliters *n*-alkane mixture and 2 mL of cell suspension were vortexed for one minute in glass tubes and equilibrated for 30 min. The aqueous phase was carefully removed and measured for absorbance at 600 nm. Higher cell surface hydrophobicity is indicated by a lower percentage of cells found in the aqueous phase.

#### PCR amplification of the *lasI/lasR* region

Genomic DNAs from *Pseudomonas aeruginosa* PA01 and ATCC 33988 were isolated using the Wizard Genomic DNA Purification Kit (Promega, Madison, WI, USA). A blastn comparison using WebACT (Imperial College, London, UK) was generated between the complete genome of *P. aeruginosa* PA01 (NC_002516) and the 209 draft sequence contigs for *P. aeruginosa* ATCC 33988. The alignment was viewed with the DNA sequence comparison viewer, Artemis Comparison Tool Release 13.0.0 (ACT, Welcome Trust Sanger Institute, Cambridge, UK). The *lasI* (PA1432) and *lasR* (PA1430) nucleotide sequences were exported from *P. aeruginosa* PA01 along with aligned flanking regions (25,510–30,561 bp) from *P. aeruginosa* ATCC 33988 contig52. The following primers were designed in Primer3Plus release 2.3.6 to validate the absence of the region containing the quorum sensing transcriptional regulators *lasI*/*lasR* in the *P. aeruginosa* ATCC 33988 strain; PA1430_lasR_F (ACGGTCAGTCACTGTACCCA), PA1430_lasR_R (TGCTGACCGGATGTTCGAAG), PA1432_lasI_F (CTCGATACCACTGGCCCCTA), PA1432_lasI_R (CGTCTGGATGTCGTTCTGCA), 33988.c52.flank_F (GTCTATCGCACCACCCACAG), 33988.c52.flank_R (TCGCCGAACTGGAAAATGG).

PCR amplification was carried out in 50 μl reaction volumes with Advantage GC 2 PCR kit (Takara Bio USA, Inc, Mountain View, CA, USA), containing 0.2 mM (each) deoxynucleoside triphosphates, 0.2 uM forward and reverse primers, and 5 ng genomic DNA. Amplification was carried out on an ABI 9700 thermal cycler. After an initial denaturization for 1 min at 95 °C, 25 cycles were completed, each consisting of 30 s at 95 °C, 30 s at 57 °C, and 60 s at 68 °C, followed by a final extension of 3 min at 68 °C. PCR amplicons were visualized via gel electrophoresis on 1.2% agarose gels.

## Additional files

Additional file 1: Table S1–S4. RAST and PsDB annotations. (PPTX 38 kb)

Additional file 2: Tables S5–S8. Microarray results. (XLSX 648 kb)

Additional file 3: Tables S9–S12. RNA-seq results. (XLSX 141 kb)

Additional file 4: Tables S13–S16. Ribo-seq results. (XLSX 481 kb)

Additional file 5: Tables S17–S20. Translation efficiency results. (XLSX 529 kb)

Additional file 6: Tables S21–S24. Proteomic MS results. (XLSX 373 kb)

Additional file 7: Figure S1. Venn diagrams. Comparison of RNA-seq, ribosome footprinting and proteomics data. Venn diagrams represent the number of *Pseudomonas aeruginosa* genes that are >2-fold increased (*p* < 0.05) in (A) PAO1 when grown in glycerol or *n*-alkanes, (B) ATCC 33988 when grown in glycerol or *n*-alkanes, and (C) one strain when compared to the other during growth in glycerol. Blue circle = RNA-seq, Pink circle = ribosome footprinting, Green circle = proteomics. (D) Percentages of total analyzed genes that fall within each of four genomic expression categories during growth in glycerol. Genes are assigned to one of four categories based on whether they appear in the ‘high’ value or ‘low’ value component of the RNA-seq (Transcription/Txn) or ribosome footprint (Translation/Trans) results. (XLSX 85 kb)

Additional file 8: Figure S2. Gene expression patterns of sample *P. aeruginosa* operons. Expression profiles of genes clusters that are (A) inhibited by PAO1 in glycerol versus PAO1 in *n*-alkanes, or (B) are induced by strain PAO1 when compared to 33988, regardless of carbon source. R = total RNA, F = ribosome footprint, P = protein. If a given gene was not detected in any sample, the corresponding box was shaded grey. If a gene was present under only one condition, the log_2_(fold-change) was assigned a value of 1 or −1. (PPTX 81 kb)

Additional file 9: Figure S3. Cell surface hydrophobicity. Cultures were grown in 5% *n*-alkanes or 5% glycerol and harvested at OD_600_ = 0.8. Cells were resuspended in M9 to an OD_600_ = 1.0 and 2 mL of cells were vortexed with 500 μL *n*-alkane mixture for 1 min. After a 30 min equilibrium phase, the OD_600_ of the aqueous phase was measured. Increased cell surface hydrophobicity was seen as a decrease in the percentage of cells in the aqueous phase. * indicates *p* < 0.05 for data discussed in the text. (PPTX 76 kb)

Additional file 10: Figure S4. Expression of the “protein activator” gene Pra. Heat maps representing the fold-change in total RNA (column R), ribosome footprints (column F) and protein (column P) of *pra*. All expression values are log2-transformed. If a gene is not present in glycerol cultures, the log_2_(fold-change) is assigned the value 1. (PPTX 46 kb)

## Abbreviations

3A: ATCC 33988+*n*-alkanes
3G: ATCC 33988+glycerol
3-oxo-C_12_-HSL: N-3-oxododecanoyl-L-homoserine lactone
ADH: Alcohol dehydrogenase
ANI: Average nucleotide identity
BATH: Bacterial adhesion to hydrocarbons
C_4_-HSL: N-butyryl-DL-homoserine lactone
EIC: Extracted ion chromatography
EPS: Extracellular polymeric substance
GCxGC: Comprehensive two-dimensional gas chromatography
LC-MS: Liquid chromatography-mass spectrometry
*n*-alkanes: normal alkanes
NSAF: Normalized spectral abundance factor
PA: PAO1+*n*-alkanes
PG: PAO1+glycerol
PQS: 2-heptyl-3-hydroxy-4(1H)-quinolone
PsDB: Pseudomonas database
QS: Quorum sensing
RAST: Rapid annotation using subsystem technology
RPKM: Reads per kilobase per million reads
SLM: Spent liquid medium
