# Supplementary material for: A comprehensive multi-omics approach uncovers adaptations for growth and survival of Pseudomonas aeruginosa on n-alkanes
Source: BMC Genomics. 2017 Apr 28;18:334. doi: 10.1186/s12864-017-3708-4 (PMC5410065; doi:10.1186/s12864-017-3708-4)
Supplement: Supplementary file 7 — Venn diagrams. Comparison of RNA-seq, ribosome footprinting and proteomics data. Venn diagrams represent the number of Pseudomonas aeruginosa genes that are >2-fold increased (p < 0.05) in (A) PAO1 when grown in glycerol or n-alkanes, (B) ATCC 33988 when grown in glycerol or n-alkanes, and (C) one strain when compared to the other during growth in glycerol. Blue circle = RNA-seq, Pink circle = ribosome footprinting, Green circle = proteomics. (D) Percentages of total analyzed genes that fall within each of four genomic expression categories during growth in glycerol. Genes are assigned to one of four categories based on whether they appear in the ‘high’ value or ‘low’ value component of the RNA-seq (Transcription/Txn) or ribosome footprint (Translation/Trans) results. (XLSX 85 kb) [file 12864_2017_3708_MOESM7_ESM.pptx]

## Slide 1
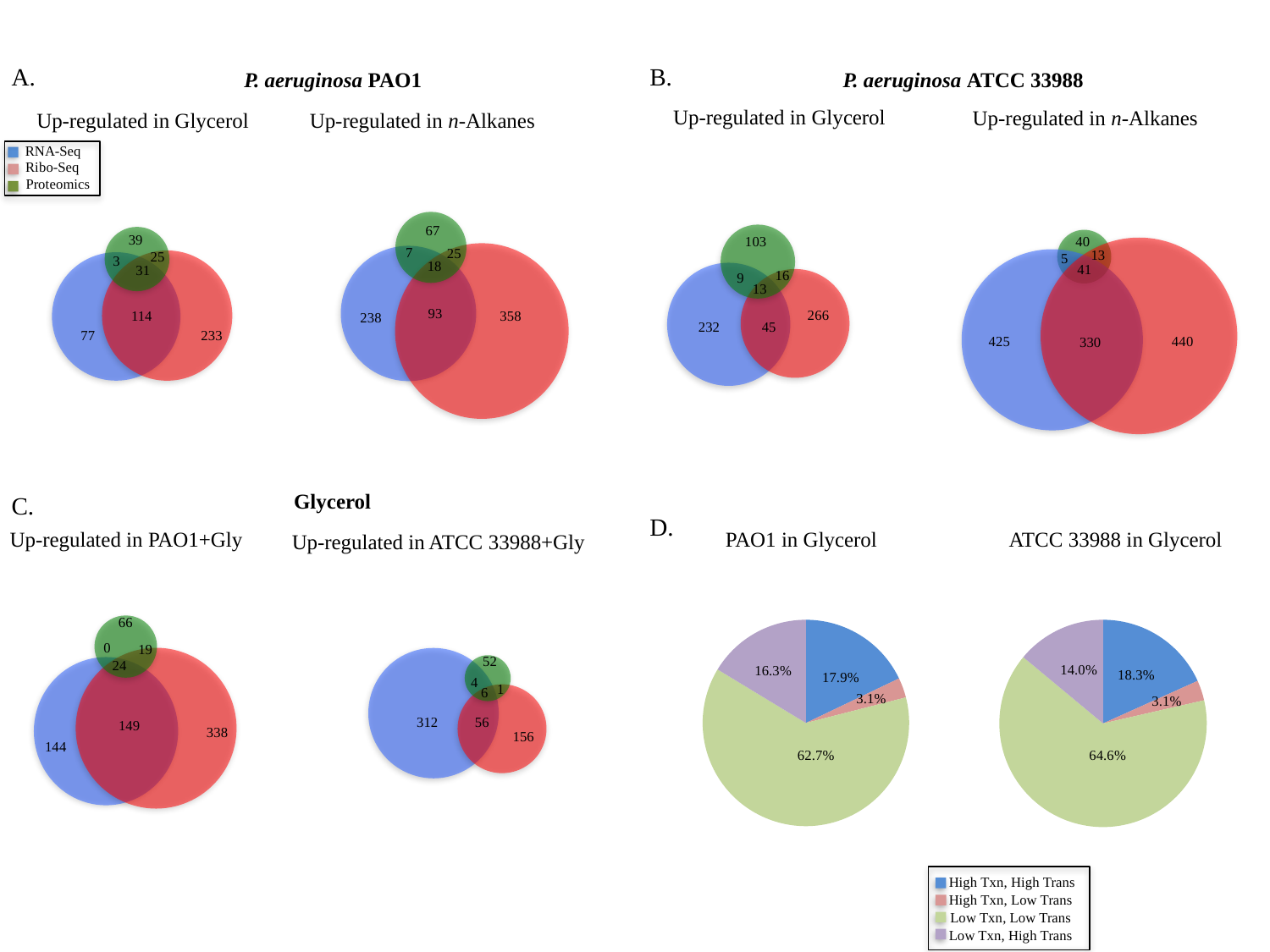

B.
A.
P. aeruginosa PAO1
P. aeruginosa ATCC 33988
Up-regulated in Glycerol
Up-regulated in n-Alkanes
Up-regulated in Glycerol
Up-regulated in n-Alkanes
RNA-Seq
Ribo-Seq
Proteomics
67
39
40
103
7
25
13
25
5
3
18
41
31
16
9
13
93
266
114
358
238
232
45
77
233
425
440
330
Glycerol
C.
D.
Up-regulated in PAO1+Gly
PAO1 in Glycerol
ATCC 33988 in Glycerol
Up-regulated in ATCC 33988+Gly
66
### Chart
| Category | |
|---|---|
| High txn, High trans | 0.179006891676975 |
| High txn, Low trans | 0.0311009012192967 |
| Low txn, Low trans | 0.626612475702421 |
| Low txn, High trans | 0.163279731401308 |
### Chart
| Category | |
|---|---|
| High txn, High trans | 0.182717794663368 |
| High txn, Low trans | 0.0314543205513342 |
| Low txn, Low trans | 0.6462272486305 |
| Low txn, High trans | 0.139600636154798 |0
19
52
24
14.0%
16.3%
18.3%
17.9%
4
1
6
3.1%
3.1%
312
56
149
338
156
144
62.7%
64.6%
High Txn, High Trans
High Txn, Low Trans
Low Txn, Low Trans
Low Txn, High Trans
