# Supplementary material for: A comprehensive multi-omics approach uncovers adaptations for growth and survival of Pseudomonas aeruginosa on n-alkanes
Source: BMC Genomics. 2017 Apr 28;18:334. doi: 10.1186/s12864-017-3708-4 (PMC5410065; doi:10.1186/s12864-017-3708-4)
Supplement: Supplementary file 8 — Gene expression patterns of sample P. aeruginosa operons. Expression profiles of genes clusters that are (A) inhibited by PAO1 in glycerol versus PAO1 in n-alkanes, or (B) are induced by strain PAO1 when compared to 33988, regardless of carbon source. R = total RNA, F = ribosome footprint, P = protein. If a given gene was not detected in any sample, the corresponding box was shaded grey. If a gene was present under only one condition, the log2(fold-change) was assigned a value of 1 or −1. (PPTX 81 kb) [file 12864_2017_3708_MOESM8_ESM.pptx]

## Slide 1
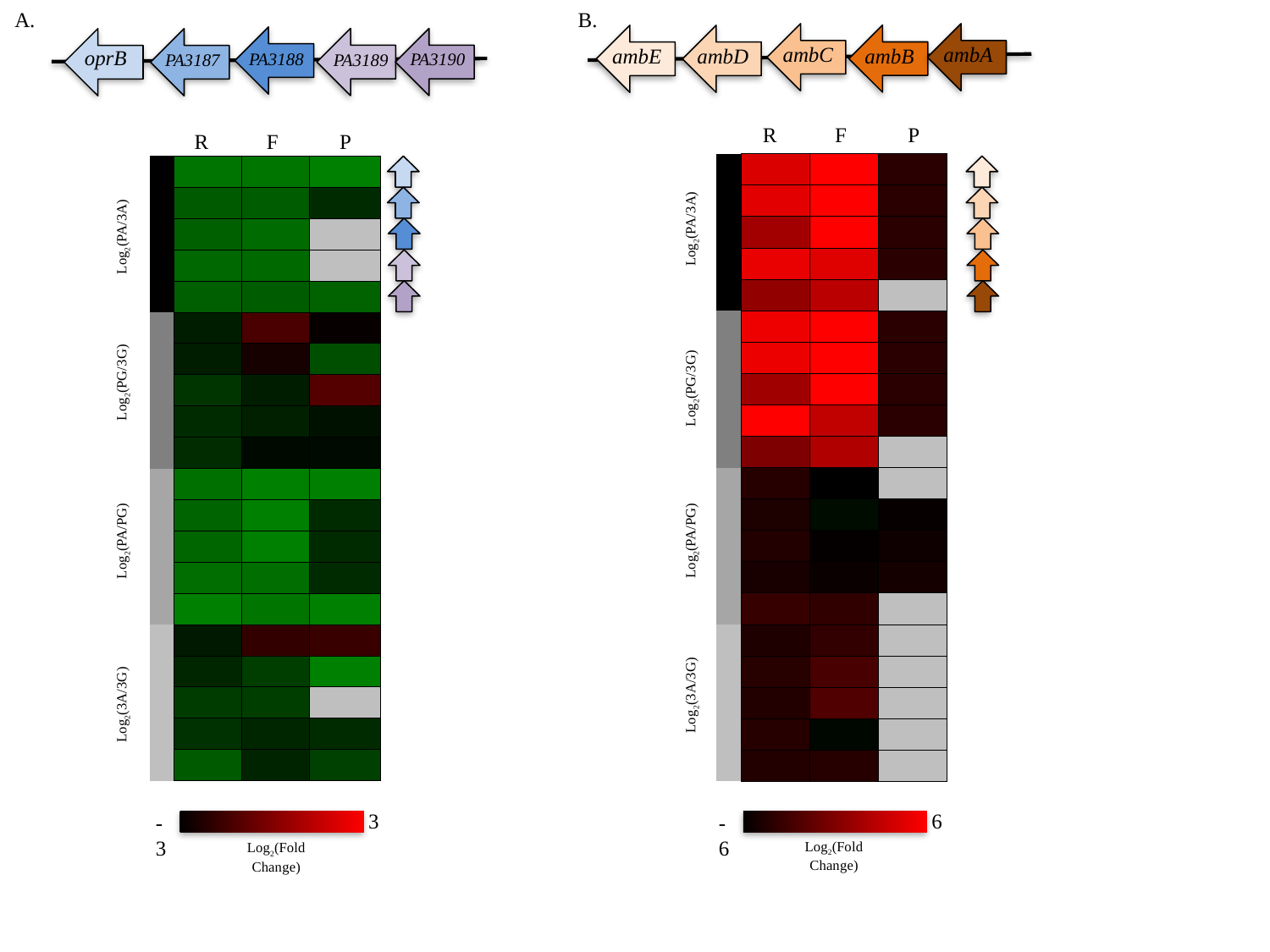

A.
B.
ambC
ambA
ambB
ambE
ambD
oprB
PA3188
PA3190
PA3187
PA3189
F
P
R
F
P
R
| | | | |
| --- | --- | --- | --- |
| | | | |
| | | | |
| | | | |
| | | | |
| | | | |
| | | | |
| | | | |
| | | | |
| | | | |
| | | | |
| | | | |
| | | | |
| | | | |
| | | | |
| | | | |
| | | | |
| | | | |
| | | | |
| | | | |
| | | | |
| --- | --- | --- | --- |
| | | | |
| | | | |
| | | | |
| | | | |
| | | | |
| | | | |
| | | | |
| | | | |
| | | | |
| | | | |
| | | | |
| | | | |
| | | | |
| | | | |
| | | | |
| | | | |
| | | | |
| | | | |
| | | | |
Log2(PA/3A)
Log2(PA/3A)
Log2(PG/3G)
Log2(PG/3G)
Log2(PA/PG)
Log2(PA/PG)
Log2(3A/3G)
Log2(3A/3G)
3
-3
Log2(Fold Change)
6
-6
Log2(Fold Change)
