# Supplementary figures and images for: A comprehensive multi-omics approach uncovers adaptations for growth and survival of Pseudomonas aeruginosa on n-alkanes
Source: BMC Genomics. 2017 Apr 28;18:334. doi: 10.1186/s12864-017-3708-4 (PMC5410065; doi:10.1186/s12864-017-3708-4)

## Slide 1
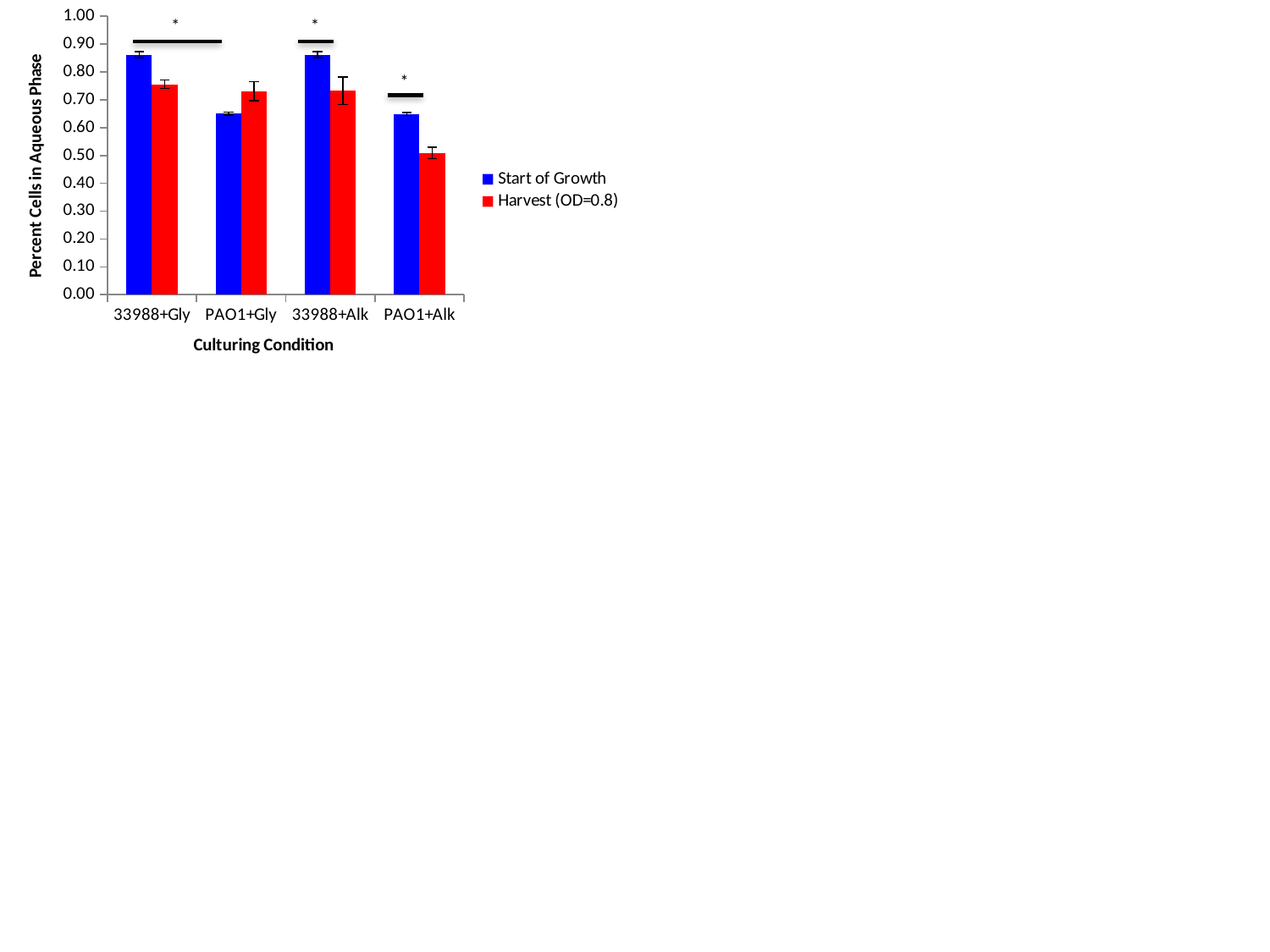

### Chart
| Category | | |
|---|---|---|
| 33988+Gly | 0.862179487179487 | 0.756031278490778 |
| PAO1+Gly | 0.651090342679128 | 0.731023921305611 |
| 33988+Alk | 0.862179487179487 | 0.732275705981317 |
| PAO1+Alk | 0.649532710280374 | 0.509477577438743 |*
*
*

Supplement: Supplementary file 9 — Cell surface hydrophobicity. Cultures were grown in 5% n-alkanes or 5% glycerol and harvested at OD600 = 0.8. Cells were resuspended in M9 to an OD600 = 1.0 and 2 mL of cells were vortexed with 500 μL n-alkane mixture for 1 min. After a 30 min equilibrium phase, the OD600 of the aqueous phase was measured. Increased cell surface hydrophobicity was seen as a decrease in the percentage of cells in the aqueous phase. * indicates p < 0.05 for data discussed in the text. (PPTX 76 kb) [file 12864_2017_3708_MOESM9_ESM.pptx]

## Slide 1
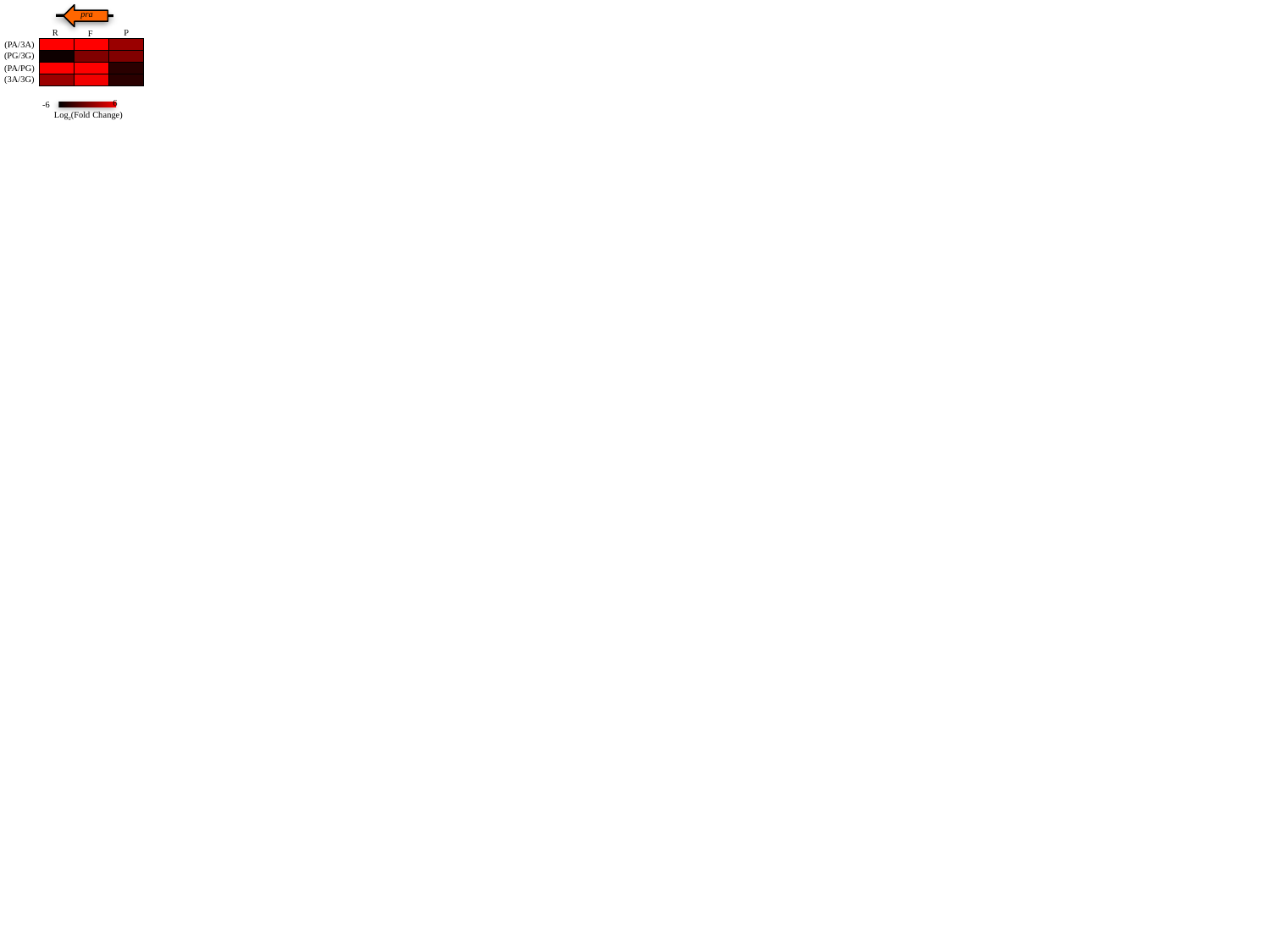

pra
P
R
F
(PA/3A)
| | | |
| --- | --- | --- |
| | | |
| | | |
| | | 1 |
(PG/3G)
(PA/PG)
(3A/3G)
6
-6
Log2(Fold Change)

Supplement: Supplementary file 10 — Expression of the “protein activator” gene Pra. Heat maps representing the fold-change in total RNA (column R), ribosome footprints (column F) and protein (column P) of pra. All expression values are log2-transformed. If a gene is not present in glycerol cultures, the log2(fold-change) is assigned the value 1. (PPTX 46 kb) [file 12864_2017_3708_MOESM10_ESM.pptx]
